# Ultrasound assisted extraction enhances phytochemical profile and functional properties of moringa leaf extract with protection against gentamicin induced nephrotoxicity

**DOI:** 10.1038/s41598-025-27520-w

**Published:** 2025-11-26

**Authors:** Asmaa M. Shehata, Sanaa M. Abdel-Hameed, Aliaa F. Anter, Rokaia R. Abdelsalam

**Affiliations:** 1 Food Science Department, Faculty of Agriculture, Minia University, Minya, 61519 Egypt; 2 Pharmacology and Toxicology Department, Faculty of Pharmacy, Minia University, Minya, 61519 Egypt

**Keywords:** Bioactive components, GC–MS, HPLC, Superoxide dismutase (SOD), Gentamicin (GN), Biochemistry, Biotechnology, Chemical biology, Chemistry, Drug discovery, Plant sciences

## Abstract

*Moringa oleifera* is a rich source of therapeutic bioactive compounds, which may protect renal function against gentamicin (GN) induced toxicity. This study applied green hydroethanolic extraction utilizing 50% (MU-50) and 70% (MU-70) to obtain bioactive compounds from Moringa leaves. The extracts were characterized and quantified using Fourier transform infrared (FTIR), Gas Chromatography–Mass Spectrometry (GC–MS), and High-Performance Liquid Chromatography (HPLC). Additionally, this study investigated their hepato-renal protection against gentamicin toxicity alongside their suitability for orange juice fortification. MU-50 exhibited stronger antioxidant activity (IC_50_ = 46.72 µg/mL) and higher phenolic (15.42 ± 0.9 mg GAE/g) and flavonoid (107 ± 0.07 µg QE/g) content compared to MU-70. FTIR analysis identified functional groups such as phenols, alkanes, ethers, esters, aromatic compounds, C–Br, and nitro compounds. GC–MS analysis identified several compounds for the first time in MU-50, including 9-oxabicyclo (3,3,1) nonan-2-one desulphosinigrin and 2-aminoethanethiol hydrogen sulfate. HPLC revealed higher concentrations of nineteen key phenolic compounds in MU-50, including chlorogenic acid, pyrocatechol and gallic acid, compared to MU-70. An in vivo study demonstrated that MU-50 at 400 ppm effectively reduced urea, creatinine, malondialdehyde (MDA), and nitrite levels in both the kidney and liver, while also restoring superoxide dismutase (SOD) activity, compared to the gentamicin group. Additionally, it significantly improved (*p* > 0.05) the physicochemical and phytochemical parameters, as well as microbial stability, while maintaining sensory acceptability in orange juice. The results highlighted that incorporating these eco-friendly hydroethanolic extracts could be a strategic move for food and beverage manufacturers as natural therapeutic agents against drug-induced toxicity*.*

## Introduction

*Moringa oleifera,* "miracle tree," is a very nutritious plant found in the Indian subcontinent. It contains vital vitamins, minerals, and bioactive components. Moreover, it has an abundance of antioxidants, antimicrobials, and anti-inflammatory effects^1,2^. Thus, *Moringa oleifera* leaves have gained attention as a potential cure for several diseases, including diabetes, hypertension, and cardiovascular diseases^3–5^. Consequently, it has been a major focus in the domains of natural medicine, pharmacology, and nutrition^6^.

On the other hand, gentamicin (GN) is widely used as a broad-spectrum antibiotic for treating severe bacterial infections caused by gram-negative bacteria. Meanwhile, the kidneys build up gentamicin and cause oxidative stress in cells^7,8^. Furthermore, prolonged use of gentamicin is associated with significant cell damage, including nephrotoxicity and hepatotoxicity^9,10^. Complementary treatments are of critical importance to decrease the harmful effects of gentamicin upon extensive usage and the presence of hazards^11^.

According to recent research, moringa extract may have nephroprotective and liver-protective effects by lowering inflammation and oxidative stress, thereby offsetting the negative impacts of gentamicin^12–14^. This positions moringa not only as a health-promoting food^15^. Thus, it has been widely investigated for its phytochemical and pharmacological potential, such as a potential adjunct therapy to reduce the toxicity associated with gentamicin. However, no previous study has comprehensively integrated green extraction optimization, multi-technique phytochemical profiling, and practical food application within a single framework. The present work uniquely combines ultrasound-assisted hydroethanolic extraction to enrich phenolic compounds, detailed FTIR, GC–MS, and HPLC characterization, and dual evaluation of both in vivo protective efficacy against gentamicin-induced nephrotoxicity and functional incorporation into orange juice. The promise of Moringa extracts as sustainable functional ingredients with therapeutic relevance is underscored by this innovative integrated approach, which bridges the gap between laboratory-scale bioactivity and real food systems.

## Materials and methods

### Chemicals and reagents

All chemicals and reagents used in this study were of analytical grade (≥ 98% purity). It was obtained from Sigma-Aldrich, Merck, and El-Naser Pharmaceutical Chemicals. The Egyptian Drug Store provided the 80 mg/2 mL ampoules of gentamicin.

### Preparation of hydro-ethanolic moringa leaf lyophilized powder extracts

*Moringa oleifera* was grown in an experimental plot of the Horticulture Research Farm, Faculty of Agriculture in Minia University, Egypt. Fresh moringa leaves (≥ 2 kg) were collected at the end of November 2023. Leaves were selected, washed, and air-dried at 35 ± 2°C for 48 h until they reached a stable weight. Following it, the dry leaves were pulverized using a laboratory mill and sieved through a 35-mesh screen (500 µm) to create a homogeneous fine powder. To protect them from light and humidity, it was stored at 4°C in amber glass containers until further analysis. Extracts were prepared following a modified method^16^ using ultrasound-assisted green extraction. The extraction duration was varied at 20, 40, 60, and 80 min under constant extraction conditions. Total phenolic yield and antioxidant activity increased up to 60 min, after which no significant improvement occurred (data not shown). Thus, 60 min was selected as the optimal time of extraction, hydro-ethanolic solutions (50% and 70%), a solid-to-solvent ratio of 1:15, and 30 ± 2°C in an ultrasonic wave single frequency (40 kHz) bath (Unique, USC-3300, Brazil). Then, extracts were vacuum-filtered by (Whatman® cotton filter), concentrated via rotary evaporator (R-300, BUCHI, Switzerland), and lyophilized for storage at − 18°C until analysis.

### Quantitation and qualification of phytochemicals

#### Determination of antioxidant activity, total phenolics, and flavonoids

The antioxidant activity of MU-50 (50%) and MU-70 (70%) hydroethanolic extracts was measured using the DPPH· assay as described by Brand-Williams et al.^17^. A 0.1 mM DPPH· methanolic solution was mixed with the extract (10–200 µg/mL) and incubated for 30 min in the dark at room temperature. Absorbance was measured at 517 nm, and ascorbic acid was used as the reference standard. The scavenging activity (%) was calculated as (A _Control_ − A _Sample_)/A _Control_ × 100, and IC_50_ values were obtained from the inhibition curve.

### Total phenolic content (TPC)

TPC was determined using the Folin-Ciocalteu method^18^. Diluted extract (0.1 mL) was mixed with Folin reagent (0.5 mL, 1:10 v/v) and 1.5 mL of 7.5% Na₂CO₃, incubated for 30 min, and measured at 765 nm. Results were expressed as mg gallic acid equivalents (GAE)/g sample.

### Total flavonoid content (TFC)

TFC was measured by the aluminum chloride colorimetric method^19^. The mixture (extract, AlCl₃, potassium acetate, and water) was incubated for 30 min and measured at 510 nm. Results were expressed as µg quercetin equivalents (QE)/g sample.

### FTIR analysis

Fourier transform infrared (FTIR) spectroscopy is thought to be a reliable and accurate way to define a functional group. FTIR analysis was performed on MU-50 and MU-70 extracts. One milligram of the sample was combined with 50 mg of KBr (FTIR-grade). Then, it was placed in an FTIR spectroscope (Shimadzu, IR Affinity, Japan), operating within a scanning range of 4000–400 cm^−1^ and with a resolution of 4 cm^−1^ ^20^.

### HPLC analysis

Phenolic and flavonoid compounds were identified and quantified by an Agilent 1260 HPLC system equipped with a diode-array detector (DAD) set at 280 nm. Component separation was performed on a Zorbax Eclipse Plus C8 column (4.6 × 250 mm, 5 µm) with a mobile phase consisting of water (A) and 0.05% trifluoroacetic acid in acetonitrile (B) at a flow rate of 0.9 mL/min. A linear gradient program was employed as follows: 82% (A) during the first minute, then decreased to 75% (A) by 11 min, followed by 60% (A) by 18 min, and finally re-equilibrated to 82% (A) over the last six minutes. The injection volume was 5 µL, and the column temperature was maintained at 40°C. The total run time was 24 min. Nineteen standard stock solutions were prepared in methanol at concentrations ranging from 0.5 to 5.0 mg/mL, depending on compound solubility. All solutions were stored at − 20°C until analysis. Calibration curves spanned concentrations from 10 to 75 µg/mL, as illustrated in Fig. 3.

### GC–MS analysis

Volatile and semi-volatile components were identified using GC–MS, which complemented the analysis of non-volatile phenolics by FTIR and HPLC, resulting in a more comprehensive chemical characterization. GC/MS analysis was carried out at the National Research Center (NRC), Giza, using a Thermo Scientific TG-5MS fused silica capillary column (30 m, 0.25 mm, 0.1 µm film thickness). The system used electron ionization (70 eV) with helium as the carrier gas at 1 mL/min. The injector and MS transfer line were maintained at 280°C. Thus, compound quantification was based on relative peak area percentages, and identification was achieved by comparing retention times and mass spectra to NIST and WILLY library data^21^.

### Animal model

The protocol of this study was reviewed and approved by the Ethical Committee of the Faculty of Pharmacy, Minia University (Approval Code: MPEC 250,504). The animal experiment was conducted at the Faculty of Pharmacology, Minia University, Egypt. All animal procedures were performed in accordance with institutional ethical standards and international guidelines. This study is reported in accordance with the ARRIVE guidelines (https://arriveguidelines.org). Twenty-four healthy mature male albino rats were obtained from Nahda University Beni-Suef (NUB), animal care unit in Egypt. Rats’ weight ranged from 160 to 200 g. Before the experiment began, the animals were acclimatized to laboratory conditions for one week under close monitoring. Rats were kept in hygienic metal cages, fed a regular meal, and given unrestricted access to potable water.

### Experimental protocol

Twenty-four male rats were randomly assigned to four groups, with six animals in each group. Treatments were administered orally via stomach tube once daily for two weeks. The ethanol extract of *Moringa oleifera* leaves was prepared at room temperature following Attah et al. (2019)^22^. Group 1 (Control) received tap water. Group 2 (GN) received glyceryl trinitrate at 50.0 mg/kg/day, as described by Khatun and Verma^23^. Group 3 (M_1_) received MU-50 extract at 200 ppm + GN, while Group 4 (M_2_) received the MU-50 extract at 400 ppm + GN. The extract doses (200 and 400 ppm) were chosen based on previously published acute and sub-chronic toxicity and pharmacological studies on *Moringa oleifera* extracts^13,24^.

### Measurement of renal and hepatic function, MDA, nitrite, and SOD activity

Twenty-four hours after the final treatment, the rats were humanely euthanized using IP injection of ketamine and xylazine at doses of 80 and 10 mg/kg, respectively. Liver and kidney samples were collected, carefully excised from each animal and promptly stored at –70°C for later biochemical and histological analysis.

Renal and hepatic functions were assessed in rats using standard assays. Serum urea and creatine levels were quantified colorimetrically^25,26^; malondialdehyde (MDA) was measured in both renal and hepatic tissues using the thiobarbituric acid assay^27^; The Griess reaction quantified nitrite levels, and absorbance was measured at 570 nm^28^; Superoxide dismutase (SOD) activity was determined by measuring the inhibition of pyrogallol autoxidation, with absorbance recorded at 420 nm^29^. Hepatic ALT and AST activities were measured spectrophotometrically using commercial diagnostic kits according to Reitman and Frankel^30^. AST and ALT colorimetric kits (CAT. No. AS 10 61 (45) and AL 10 31 (45), respectively) were provided by the Egyptian Bio-diagnostic Company, with absorbance changes monitored at 340 nm^31^. All results were expressed relative to a control group for comparison**.**

### Histopathological analysis

Liver and kidney tissues were cleared with xylene; embedded in paraffin, and fixed in 10% formalin using an automated tissue processor. Five-micron-thick sections were cut with a microtome (Leitz 1512, Leitz, Wetzlar, Germany) and stained with hematoxylin and eosin^32^. For each organ, five stained sections were examined under a microscope at varying magnifications to assess histological changes and conduct histomorphometry analys^33^. Histopathological abnormalities were graded based on five fields per section. Image J software was used to quantitatively analyze the changes before performing statistical evaluations.

### Application of functional juices

### Orange juice preparation

Orange juice was freshly extracted and subsequently blended with different concentrations of MU-50 at levels of 200 ppm and 400 ppm. Juice mixtures were transferred into sanitized, tightly sealed glass bottles and pasteurized at 90°C for 30 s in a water bath. Immediately cooling after processing. The juice samples were analyzed for various quality parameters**.**

### Physicochemical and phytochemical analyses

All physicochemical evaluations were conducted on the same day the juice was produced. It is including pH measured with a digital pH meter (model 41,250, ICMOR, USA), total soluble solids (TSS, measured with a refractometer) described by Horwitz^34^, titratable acidity^35^, vitamin C was determined by the 2,6-dichlorophenol-indophenol method^36^, β-carotene was measured using spectrophotometry as described by Nagata & Yamashita^37^ and color characteristics and intensity color difference meter (model color Tec-PCM, US)^38,39^. The antioxidant activity of the total phenolic content was assessed using a modified DPPH radical scavenging assay, as described by Abdelmegiud et al. (2024)^40^; total phenolic content^18^ and total flavonoid were determined as described by Abu Bakar et al. (2009)^41.^

### Microbiological evaluation

Microbiological evaluations included total count colony detected at 30°C in compliance with APHA (1992) methodology. The AOAC method was used for *Escherichia coli* and total coliforms on chromogenic media following incubation at 44°C and 37°C, respectively. Fungi were determined using PDA (potatoes dextrose agar)^42^.

### Sensory evaluation

Sensory evaluation was carried out to evaluate five organoleptic attributes, including flavor, odor, color, texture, and overall acceptability. A panel of 15 trained individuals was selected based on their sensory acuity and absence of biases. Using a nine-point hedonic scale, which went from 1 (strongly dislike) to 9 (strongly like), sensory attributes were scored. Each panelist was given 25 ml of juice supplied in a transparent, white glass for easy visual examination. Water was available to clear the palate in between samples^43^.

### Statistical analysis

Statistical analysis took place using Prism software. Results are shown as arithmetic means plus or minus standard deviation (SD). Duncan’s multiple range test was applied at a significance level of 5%. The IR Analyzer Spectroscopic solution software was used to analyze the FTIR spectra, making it easier to identify and assign functional groups based on peak positions and intensities.

## Results and discussion

Several studies to date have explored individually either antioxidant properties, ultrasound-assisted extraction, or phytochemical composition of *Moringa oleifera* leaves ^20,44–50^. Our work is distinctive in integrating ultrasound-assisted hydroethanolic extraction, comprehensive FTIR–GC–MS–HPLC profiling, in vivo nephro-hepatoprotection, and functional orange juice fortification. This integrated design strengthens the translational relevance of *Moringa oleifera* as both a therapeutic and functional food ingredient.

### Phytochemical characterization and antioxidant activity of moringa leaf extracts

Moringa leaf extracts exhibited a rich phytochemical profile comprising multiple bioactive compounds that varied according to plant type, environment, and nutritional factors. The enhanced bioactivity of the MU-50 extract can first be attributed to the ultrasound-assisted extraction process. Ultrasonic cavitation generates localized high-pressure and temperature microbubbles that disrupt plant cell walls, increasing solvent penetration and accelerating the release of intracellular phenolics. The use of 50% hydro-ethanol provides an optimal polarity balance, allowing efficient recovery of both polar and moderately non-polar phenolics^51,52^. This explains the higher total phenolic and flavonoid contents obtained for MU-50 compared with MU-70, shown in Table 1. At the same time, the higher ethanol fraction may have reduced cavitation intensity and the extraction of hydrophilic compounds. The following DPPH· radical scavenging assay revealed that MU-50 exhibited a lower IC_50_ value (46.72 µg/mL) than MU-70 (53.02 µg/mL). It was indicating superior antioxidant capacity and suggesting possible hepatoprotective and nephroprotective benefits supported by Drăgoi (2024)^52^ and Chandimali (2025)^53^.

**Table 1:** Phytochemicals and antioxidant activity of moringa leaf extracts

Extract	TP (mg GAE/g)	TF (µg QE/g)	DPPH (%)	IC_50_ (µg/mL)
MU-50	15.4 ± 0.9^a^	107^a^ ± 0.07^a^	75.3 ± 0.03^a^	46.72
MU-70	11.0 ± 1.1^b^	77.6 ± 0.04^b^	69.3 ± 0.08^b^	53.02

TP: total phenols, TF: total flavonoids; The data were obtained from three triplicate measurement (n = 3) and presented as the mean ± standard deviation (SD). The statistical significances are denoted as *p > 0.05*.

### Qualification of functional groups via FTIR

**Fig. 1:**
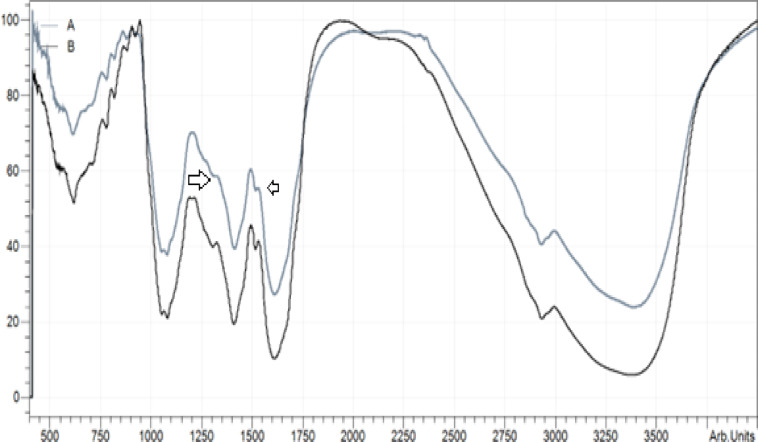
FTIR chromatogram demonstrating the functional groups of A: MU-50 and B: MU-70

FTIR was utilized to define a major functional group of Moringa oleifera leaf extracts that contribute to the antioxidant and anti-inflammatory characteristics, as shown in Fig. 1. Comparative FTIR spectra of MU-50 and MU-70 revealed similar functional group profiles but clear differences in band intensities, indicating variation in compound abundance. MU-50 exhibited a stronger and broader O–H stretching band around 3350–3380 cm^−1^, reflecting higher levels of hydroxylated phenolics and hydrogen bonding with (73%) more intensity. The aromatic C=C and C–N stretching peaks at 1606–1408 cm^−1^ and the C–O stretching bands between 1077–1038 cm^−1^ were also more intense (50%) in MU-50, consistent with its greater phenolic^20,54^, alcohols^55^, acids, esters^56^, and flavonoid content ^57^. Aliphatic bromo compounds are less common in plant matrices. They showed weak C–Br stretching in the 616.9–548.1 cm⁻^1^ region. In contrast, MU-70 showed relatively stronger C–H stretching vibrations at 2932–2850 cm⁻^1^, suggesting a higher proportion of less polar aliphatic or lipid-derived compounds^58^. These spectral variations corroborate the quantitative results, confirming that the 50% hydroethanolic solvent favored the extraction of polar phenolic compounds, while higher ethanol content enriched more nonpolar constituents.

### Identification of bioactive compounds by GC–MS

**Fig. 2:**
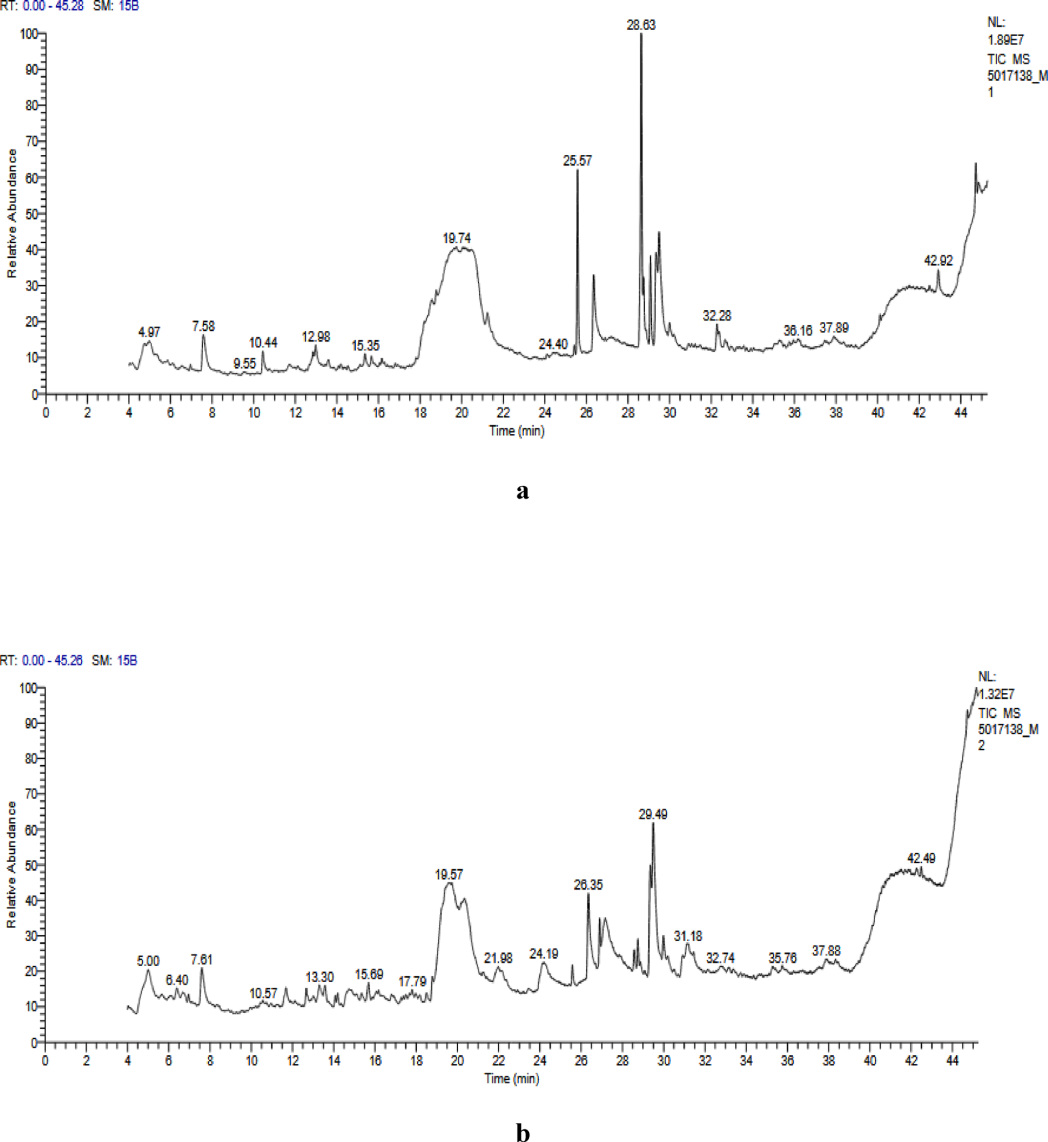
(**a**) GC-MS chromatogram of MU-50, demonstrating the compound’s retention time. (**b**) GC-MS chromatogram of MU-70, demonstrating the compound’s retention time

Moringa oleifera hydro-ethanolic extracts contained 37 various compounds in the MU-50 and 30 components in MU-70 extracts (Tables 2 and 3). These compounds were identified by matching their mass spectra to data from the NIST/Wiley database, as illustrated in Fig. 2a and b. The thirty-seven peaks in the GC–MS chromatograms indicated a diverse range of phytochemicals such as flavonoids, phenolic acids, and alkaloids. That may provide valuable insights for future therapeutic applications. The MU-50 extracts contained 9-oxabicyclo[3.3.1]nonan-2-one and the flavonoid derivative (4H-pyran-4-one), which are known as antibacterial and anti-inflammatory ^59^. Other notable compounds were investigated, including cis-10-heptadecenoic acid and 7,10,13-hexadecatrienoic acid, which have anticancer and antioxidant properties^60^. Additionally, glucosinolate desulphosinigrin was detected for the first time in MU-50, which has an anti-asthmatic effect^61^. D-mannose, glyceryl 1-caprate, and palmitoleic acid were investigated in both of the extracts, which were linked to antidiabetic, anti-inflammatory, and antimicrobial activities^62–65^.

**Table 2:** GC-MS profiling of phytochemicals in MU-50 hydro-ethanolic extract

PN	Compound	MF	MW (g/mol)	Area (%)	Compound Sub-class
1	4H-Pyran-4-one, 2,3-dihydro-3,5-dihydroxy-6-methyl	C_6_H_8_O_4_	144	3.35	Flavonoid
2	9-Oxabicyclo [3.3.1] nonan-2-one, 5-hydroxy	C_8_H_12_O_3_	156	1.72	Lactone
3	Methyl 13C hexadecatrienoate	C_17_H28O_2_	264	1.11	Carotenoid Derivatives
4	7,10,13-Hexadecatrienoic acid, methyl ester	C_17_H_28_O_2_	264	1.56	Fatty acids
5	1H-indol-5-ol, 3-(2-aminoethyl)	C_10_H_12_N_2_O	176	0.67	Indole alkaloids.
6	Cyclohexane, 1R-acetamido-4cis-acetoxy-5,6Zcisep, oxy-2cis,3trans-dimethoxy-	C_12_H1_9_NO_6_	273	1.01	Alkaloid
7	4-(2,4,4-Trimethyl-cyclohexa-1,5-dienyl)-but-3-en-2-one	C_13_H_18_O	190	0.63	Terpenoids
8	10-Heptadecen-8-ynoic acid, methyl ester, (E)-	C_18_H_30_O_2_	278	0.63	Fatty acid ester
9	5,5,8a-Trimethylhexahydro-2H-chromen-4a(5H)-yl acetate	C_14_H_24_O_3_	240	0.68	Lactone
10	2-Aminoethanethiol hydrogen sulfate (ester)	C_2_H_7_NO_3_S_2_	157	0.95	Sulfonate Ester
11	Desulphosinigrin	C_10_H_17_NO_6_S	279	1.20	Thio-glycosides
12	D-Mannose	C_6_H_12_O_6_	180	1.35	Aldohexose
13	α-D-Glucopyranose, 4-O-α-D-galactopyranosyl-lactose	C_12_H_22_O_11_	342	0.72	Glycosides
14	Melezitose	C_18_H_32_O_16_	504	1.42	Trisaccharide
15	Glyceryl 1-caprate	C_13_H_26_O_4_	246	1.25	Monoacylglycerol
16	Palmitoleic acid	C_16_H_30_O_2_	254	2.25	SFA
17	9-Hexadecenoic acid, methyl ester, (Z)	C_17_H_32_O_2_	268	0.57	MUFA
18	Hexadecanoic acid, methyl ester	C_17_H_34_O_2_	270	10.8	Fatty Acyls.
19	n-Hexadecanoic acid	C_16_H_32_O_2_	256	5.22	SFA
20	Ethyl 9,12,15-octadecatrienoate	C_16_H_32_O_2_	306	20.8	USFA ester
21	10-Octadecenoic acid, methyl ester	C_19_H_36_O_2_	296	3.16	MUFA
22	Phytol	C_20_H_40_O	296	5.47	Diterpene alcohol
23	9,12-Octadecadienoic acid (Z, Z)	C_18_H_32_O_2_	280	7.95	Fatty acid
24	cis-Vaccenic acid	C_18_H_34_O_2_	282	7.80	MUFA
25	Dasycarpidan-1-methanol, acetate (ester)	C_20_H_26_N_2_O_2_	326	1.24	Indole alkaloids
26	6,9,12,15-Docosatetraenoic acid, methyl ester	C_23_H_38_O_2_	346	2.12	PUFA
27	1a,2,5,5a,6,9,10,10a-octahydro-5,5a,6-trihydroxy-1,4-bis(hydroxymethyl)-1,7,9-trimethyl	C_20_H_28_O_6_	364	0.76	Terpenoids
28	6,9,12-Octadecatrienoic acid, methyl ester	C_19_H_32_O_2_	292	0.52	Fatty acid ester
29	Arachidonoyl Ethanolamine	C_22_H_37_NO_2_	347	0.52	Fatty acid amide
30	1-Heptatriacotanol	C_37_H_76_O	536	0.49	Fatty alcohol
31	(2,3-Bis[(trimethylsilyl)oxy] propyl (9Z,12Z)-9,12-octadecadienoate	C_27_H_54_O_4_Si_2_	498	0.53	Diphenylmethanes
32	Arabinitol	C_15_H_22_O_10_	737	0.50	Sugar alcohol
33	Quercetin7,3′,4′-trimethyl ether	C_18_H_16_O_7_	344	0.81	Fatty acid ester
34	2-(5-Hydroxypent-2-ynyl)-3-oxocyclopentyl] thioacetal acid, S-t-butyl ester	C_16_H_24_O_3_S	296	1.89	Phenols
35	Psi.,.psi.-Carotene, 1,1',2,2'-tetrahydro-1,1'-dimethoxy-	C_42_H_64_O_2_	600	3.25	Carotenoids
36	α-D-Galactopyranoside, methyl2,6-bis-O-(trimethylsilyl), cyclic	C_17_H_37_BO_6_Si_2_	404	2.29	Glycosides
37	2,3-Bis[(trimethylsilyl)oxy] propyl (9Z,12Z)-9,12-octadecadienoate	C_27_H_54_O_4_Si_2_	762	2.29	Fatty acid ester

RT: retention time, PN: peak number, MF: molecular formula, MW: molecular weight

**Table 3:** GC-MS profiling of phytochemicals in MU-70 hydro-ethanol extract

PN	Compounds	MF	MW (g/mol)	Area (%)	Compound Sub-class
1	3,4,5,6-Tetrahydroxy-2-oxo-hexanoic	C_6_H_10_O_7_	194	2.31	L-ketoidonate
2	2-(5-Methyl-5-vinyltetrahydro-2-furanyl)-2-propanol	C_10_H_18_O_2_	170	1.52	Terpenoid
3	4H-Pyran-4-one, 2,3-dihydro-3,5-dihydroxy-6-methyl-	C_6_H_8_O_4_	144	4.24	Flavonoid
4	3,5-Heptadienal, 2-ethylidene-6-methyl-	C_10_H_14_O	150	1.54	Monoterpenoid
5	Naphthaliene, 1,2-dihydro-1,5,8-trimethyl-	C_13_H_16_	172	1.58	Alkylnaphthalene
6	9- Octadecenamide	C_18_H_35_NO	281	2.32	Fatty acid amide
7	l-Gala-l-ido-octonic lactone	C_8_H_14_O_8_	238	1.56	Cyclic ester
8	9-Heptadecene-4,6-diyn-8-ol, (Z)-	C_17_H_26_O	246	1.18	Alkynol
9	Cedran-diol, 8S,13-	C_15_H_26_O_2_	238	1.98	Sesquiterpenoid
10	Cyclopenta 1,3]cyclopropa[1,2]cyclohepten-3(3aH)-one, 1,2,3b,6,7,8-hexahydro-6,6-dimethyl-	C_13_H_18_O	190	1.35	Sesquiterpenoid
11	D-Mannose	C_6_H_12_O_6_	180	5.85	Aldohexose
12	Ethyl α-d-glucopyranoside	C_8_H_16_O_6_	208	1.78	alkyl glycoside
13	Hexopyranosyl-(1->3) hex-2-ulofuranosyl hexopyranoside	C_18_H_32_O_16_	504	2.97	Trisaccharide
14	α - D-Glucopyranose, 4-O- α -D-galactopyranosyl	C_12_H_22_O_11_	342	4.51	O-glycoside
15	Melezitose	C_18_H_32_O_16_	504	4.15	Trisaccharide
16	3-O-hexopyranosylhex-2-ulofuranosyl	C₁₈H₃₂O₁₆	342	3.04	Trisaccharide
17	Glycerol 1-palmitate	C_19_H_38_O_4_	330	3.41	Glycero-lipid
18	Methyl 9,10-octadecadienoate	C_19_H_34_O_2_	302	2.13	Fatty acid ester
19	n-Hexadecanoic acid	C_16_H_32_O_2_	256	7.54	Fatty acid
20	Octadecadienoic acid, ethyl ester	C_20_H_40_O_2_	312	2.67	Fatty acid ester
21	Pentadecanoic acid (PDA, C15:0)	C_15_H_30_O_2_	242	4.81	SFA
22	[1,1'-Bicyclopropyl]-2-octanoic acid, 2'-hexyl-, methyl ester	C_21_H_38_O_2_	322	1.80	Fatty acid ester
23	10-Octadecenoic acid, methyl ester	C_19_H_36_O_2_	296	2.47	Fatty acid ester
24	9,12-Octadecadienoic acid (Z, Z)- linoleic	C_18_H_32_O_2_	280	10.5	Fatty acid ester
25	cis-Vaccenic acid	C_18_H_34_O_2_	282	12.9	Ester
26	Dasycarpidan-1-methanol, acetate (ester)	C_20_H_26_N_2_O_2_	326	2.27	Terpene Ester
27	1,25-Dihydroxyvitamin D3, TMS Derivative	C_30_H_52_O_3_Si	488	1.46	Steroids
28	Octadecanoic acid, (2-phenyl-1,3-dioxolan-4-yl) methyl ester, cis	C_28_H_44_O_4_	444	1.08	Fatty acid ester
29	H-1-benzopyran-4-one, 2-(3,4-dimethoxyphenyl)-3,5-dihydroxy-7-methoxy	C₁₈H₁₆O₇	344	0.80	Flavone
30	Arabinitol, pentaacetate	C_29_H_33_ClN_2_O_2_	476	1.92	Sugar Alcohol

RT: retention time, PN: peak number, MF: molecular formula, MW: molecular weight

The phytochemical profile provided additional depth information about MU-70 extracts. Phytol, a diterpene alcohol, is present in both extracts in good proportions. It highlights the structural backbone as a terpene derivative and notes its use in synthesizing vitamins E and K_1_. Additionally, it has antioxidant, anti-inflammatory, antimicrobial, and anticancer properties^65^. Furthermore, the C–H stretching bands observed in FTIR (indicative of aliphatic CH₂/CH₃ groups) in MU-70. These were reflected in the GC–MS detection of fatty acids such as palmitic, oleic, and linoleic acids. They are well known for their anti-inflammatory effect and support heart health^66^. Similarly, the C–N stretching bands in FTIR aligned with nitrogen-containing alkaloids and glucosinolates identified via GC–MS, highlighting the contribution of aromatic amines and other nitrogenous compounds.

Additionally, 3,4,5,6-Tetrahydroxy-2-oxo-hexanoic, 2-(7-heptadecynyloxy) tetrahydro-2H-pyran, and 2-furanmethanol were detected and have shown strong effects against cancer and microbes^67^. Moreover, MU-70 contained unique molecules such as 1,25-dihydroxyvitamin D_3_ and pentadecanoic acid, which are reported to have bone health benefits and cardiovascular^68,69^. Furthermore, the presence of deoxyspergualin and quercetin derivatives highlighted the immune-modulatory and antioxidant potential of these extracts. So, it may be used for medical applications. The co-occurrence of these compounds and functional group signatures demonstrates a direct link between the vibrational features detected by FTIR and the actual phytoconstituents present in the extracts.

### Characterization of phenolic and flavonoid by HPLC

**Fig. 3:**
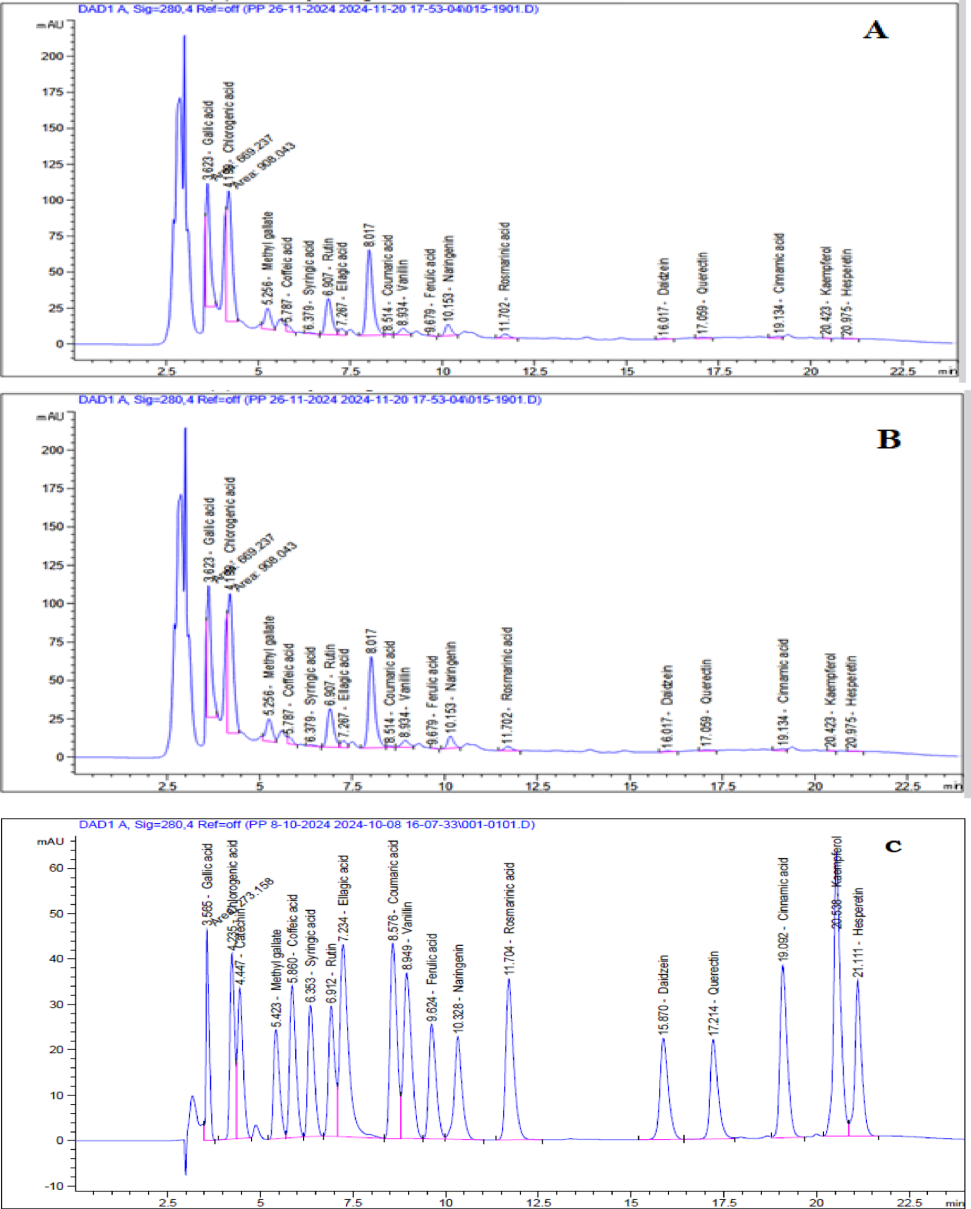
HPLC Chromatograms of phenolic and flavonoid of (**A**) MU-50, (**B**) MU-70 and standard (**C**)

Phenolic components and flavonoids were identified and quantified in hydroethanolic extracts of *Moringa oleifera* leaves using HPLC (Table 4 and Fig. 3a and b). Nineteen phenolic compounds were identified and quantified based on their retention periods and spectrum characteristics by comparison with standard references. Relative to MU-50, MU-70 demonstrated enhanced recovery of key phenolics, including gallic acid (1.35-fold) and pyrocatechol (1.25-fold). This pattern underscores the pivotal role of solvent polarity in modulating the extraction efficiency. These compounds are well-documented for their antioxidant, anti-inflammatory, and antidiabetic roles^70,71^. The observed variation in phytochemical composition between MU-50 and MU-70 strongly suggests that the percentage of ethanol used during extraction plays a decisive role in the selectivity and yield of individual bioactive compounds^72^.

**Table 4:** HPLC phytochemical analysis of hydro-ethanolic *Moringa oleifera* leaf extracts

PN	Compound	RT	MU-50 (%Area)	MU-70 (% Area)	MF
1	Gallic acid	3.623	21.4	29.0	C_7_H_6_O_5_
2	Chlorogenic acid	4.199	28.9	26.8	C_16_H_18_O_9_
3	Catechin	4.450	nd	0.07	C_15_H_14_O_6_
4	Methyl gallate	5.256	5.55	2.65	C_8_H_8_O_5_
5	Caffeic acid	5.787	1.25	0.8	C_9_H_8_O_4_
6	Syringic acid	6.379	0.39	0.13	C_9_H_10_O_5_
7	Rutin	6.907	9.85	4.73	C_27_H_30_O_16_
8	Ellagic acid	7.267	1.56	0.99	C_14_H_6_O_8_
9	Pyro catechol	8.010	22.8	28.7	C_6_H_6_O_2_
10	Coumaric acid	8.514	0.33	nd	C_9_H_8_O_3_
11	Vanillin	8.934	2.17	3.12	C_8_H_8_O_3_
12	Ferulic acid	9.679	0.05	0.06	C_10_H_10_O_4_
13	Naringenin	10.15	2.91	3.78	C₁₅H₁_2_O_5_
14	Rosmarinic acid	11.70	1.20	0.49	C_18_H_16_O_8_
15	Daidzein	16.02	0.28	0.10	C_15_H_10_O_4_
16	Quercetin	17.06	0.55	0.30	C_15_H_10_O_7_
17	Cinnamic acid	19.13	0.62	0.23	C_9_H_8_O_2_
18	Kaempferol	20.42	0.08	0.07	C_15_H_10_O_6_
19	Hesperetin	20.98	0.12	nd	C_16_H_14_O_6_

RT: retention time, PN: peak number, MF: molecular formula, nd: not detected

Meanwhile, MU-50 exhibited nearly twofold higher levels of rutin and methyl gallate, as well as chlorogenic acid (1.08-fold), compared to MU-70. This enrichment indicates that a lower ethanol concentration favors the extraction of more polar phenolic compounds such as flavonoid glycosides (rutin) and phenolic esters (methyl gallate), which are typically more soluble in aqueous-rich solvents. In contrast, the amounts of vanillin and naringenin in MU-70 were relatively high, approximately 1.43 and 1.3 times more than those in MU-50, respectively. Whereas, reflecting the higher solubilizing capacity of ethanol-rich systems for moderately polar or nonpolar phenolics^72,73^. This shift in phenolic profile is consistent with previous studies, which report that solvent polarity substantially influences the extraction efficiency of flavonoids and their concentrations. Specifically, semi-polar compounds are maximized at higher ethanol concentrations, whereas phenolic acids (e.g., polar compounds) are better extracted at lower ethanol concentrations. There were differences in the concentration of vanillic acid, p-coumaric, m-coumaric, ferulic, rosmarinic, kaempferol, hesperetin, and cinnamic acid compared to those given by Sukmawaty^74^. Thus, the present results highlight the importance of optimizing solvent composition to selectively enrich specific phytochemicals according to their polarity and functional relevance^24,75–77^.

### Hepatoprotective effects of *Moringa oleifera* extract in GN-induced liver injury

**Fig. 4:**
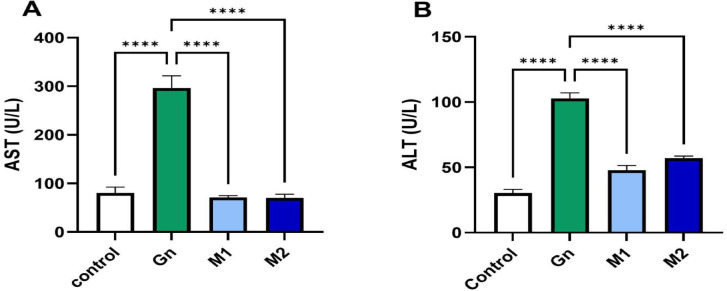
Bar chart showing the serum level of (**A**) ALT and (**B**) AST measured h post-GN administration (n=6/group). Data are represented as mean ± SEM. GN: Gentamycin M_1_: MU-50 extracts at 200 ppm; M_2_: MU-50 extracts at 400 ppm, p value < 0.05

In this study, the hepatoprotective effects of MU-50 extract were evaluated using a gentamicin (GN)-induced hepatotoxicity rat model. The GN group exhibited a marked increase (*p* < 0.05) in AST and ALT enzyme levels (Fig. 4), which is indicative of hepatocellular damage. These biomarkers were attenuated significantly (*p* < 0.05) when both treatments with MU-50 extracts at concentrations of 200 ppm (M_1_) and 400 ppm (M_2_), respectively. It had a protective effect on hepatic cellular integrity, shown in Fig. 4.

**Fig. 5:**
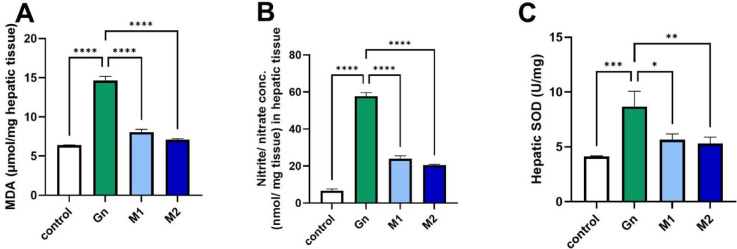
Bar chart showing the hepatic (**A**) MDA, (**B**) nitrite, and (**C**) SOD level measured post-GN administration (n=6/group). Data are represented as mean ± SEM. GN: Gentamycin, M_1_ : MU-50 extracts at 200 ppm; M_2_: MU-50 extracts at 400 ppm, p value < 0.05

The results were in line with Abo-Elmaaty^13^, who demonstrated that oral administration of moringa extract led to a substantial reduction in serum ALT and AST levels consistent with improved liver function in hepatotoxic rats. Furthermore, it significantly restored SOD activity and decreased nitrite levels, as seen in Fig. 5. So, it effectively mitigated oxidative damage and restored redox homeostasis. The elevated antioxidant activity of MU-50 corresponds to its greater content of hydroxylated phenolic compounds capable of donating hydrogen atoms or electrons to neutralize free radicals such as DPPH. The synergistic presence of phenolic acids and flavonoids enhances the total radical scavenging capacity through resonance stabilization and metal-chelating effects. Such compounds can also interrupt lipid-radical chain reactions, which contributes to the lower malondialdehyde (MDA) values observed in Fig. 5. On the other hand, GN exposure markedly increased hepatic MDA and nitrite concentrations as indicated as increased lipid peroxidation and oxidative stress, while accompanied by a significant (*p* < 0.05) decrease in the activity of SOD activity compared to the control group. These results support that MU-50 was able to prevent GN-induced liver damage by scavenging free radicals and functioning as an antioxidant^78^.

**Fig. 6:**
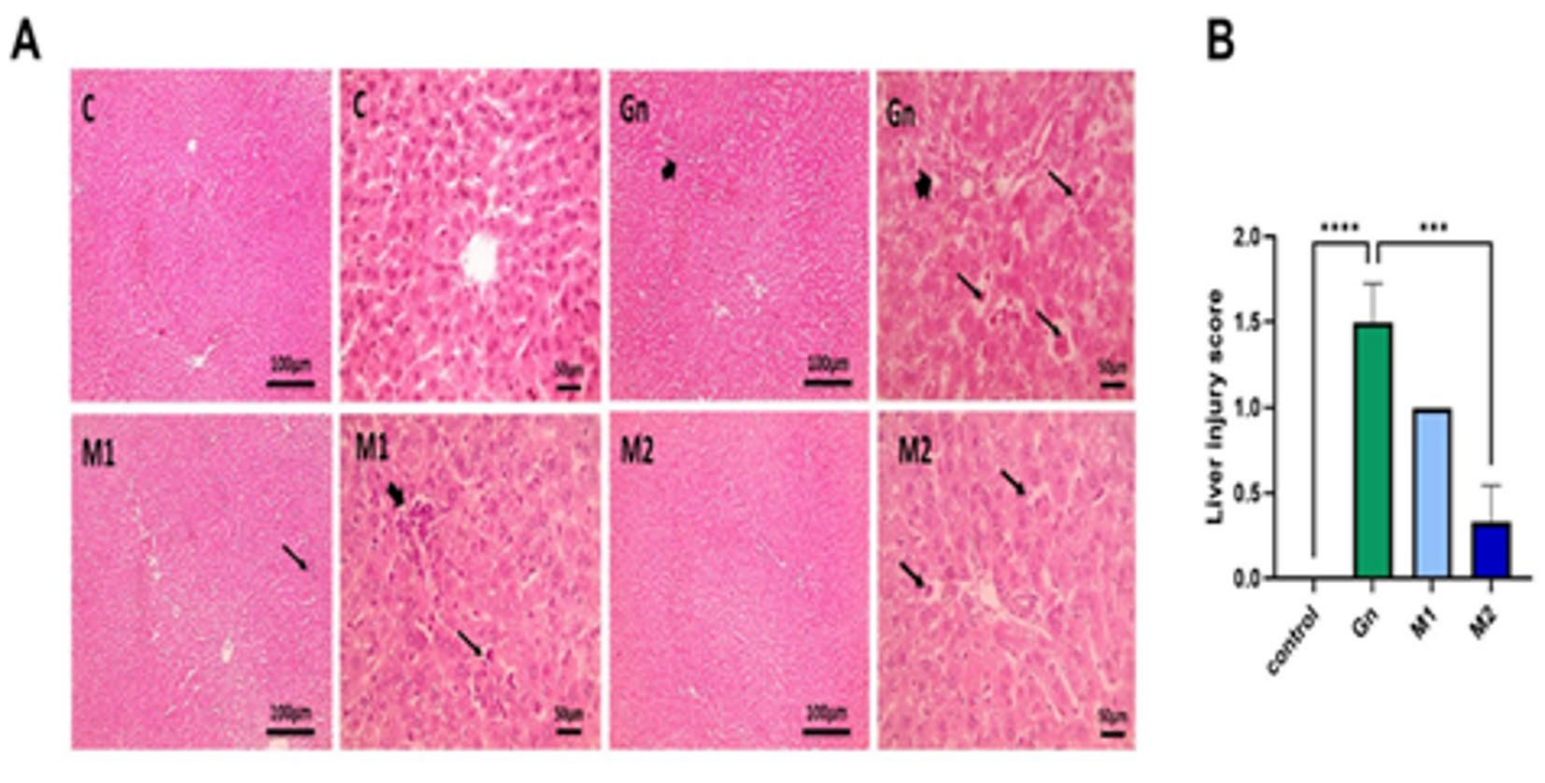
Photomicrographs of H & E-stained liver sections from control, GN, and different phenol extracts and histopathological scoring of hepatic injury. Thick black arrows: portal inflammation. Thin black arrows: extramedullary hematopoiesis in dilated sinusoids. Low magnification X: 100 bar 100 and high magnification X: 400 bar 50. Hepatic injury scores are statistically analyzed by Kruskal-Walli’s test followed by Dunn’s test to compare all means. Data are represented as mean ± SEM. Different alphabetical letters mean significant at *p* > 0.05. C: Control, Gn: Gentamycin, M: moringa, n = 6/group.

Histopathological evaluation provides an additional dimension by demonstrating that MU-50 extract has a protective effect against gentamicin (GN)-induced hepatic injury. H & E-stained liver sections showed normal hepatocytes, sinusoids, and portal areas in the control normal group (Fig. 6, C). Liver tissues from GN-treated rats displayed significant histopathological changes consistent with acute liver damage. It had pronounced portal inflammation and extensive extramedullary hematopoiesis within markedly dilated hepatic sinusoids (Fig. 6, GN). Conversely, rats treated with MU-50 extracts showed significant histological improvement, with the degree of restoration correlating with the specific extract administered. The M_1_ group exhibited mild portal inflammation and mild extramedullary hematopoiesis (Fig. 6, M_1_). Meanwhile, the M_2_ group showed a minimal histological alteration, limited to mild hematopoiesis and largely preserved hepatic architecture (Fig. 6, M2).

These protective effects were quantitatively confirmed through morphometric analysis, which revealed significantly reduced hepatic injury scores in both M_1_ and M_2_ treatment groups, with the most substantial reduction observed in the M_2_ group. Thus, *Moringa oleifera* can reduce oxidative stress, inhibit inflammatory responses, and promote both the structural and functional integrity of hepatic tissue^13^.

### Reno-protective effects of *Moringa oleifera* extract in GN-induced kidney injury

**Fig. 7:**
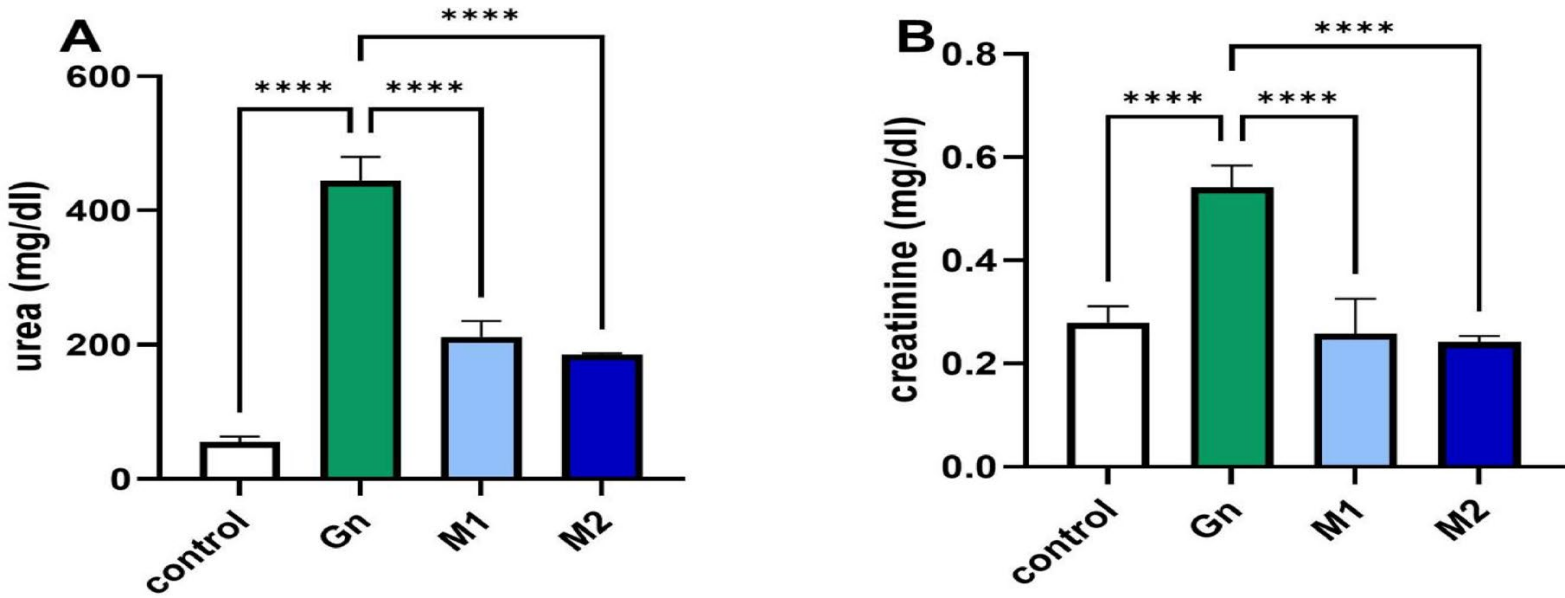
Bar chart showing serum level of (**A**) urea, and (**B**) creatinine measured h post-GN administration (n=6/group). Data are represented as mean ± SEM. GN: Gentamycin, M_1_: MU-50 extracts at 200 ppm; M_2_: MU-50 extracts at 400 ppm, p value < 0.05.

**Fig. 8:**
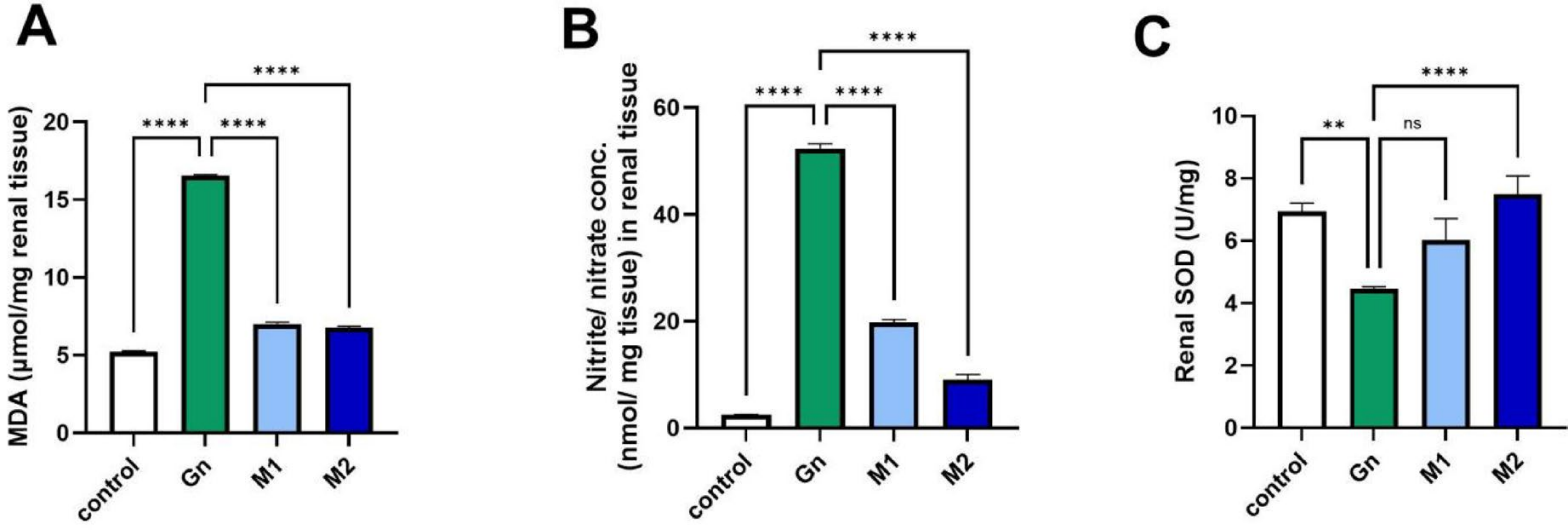
Bar chart showing the renal (**A**) MDA, (**B**) nitrate, and (**C**) SOD level measured h post-GN administration (n=6/group). Data are represented as mean ± SEM. GN: Gentamycin, M_1_: MU-50 extracts at 200 ppm; M_2_: MU-50 extracts at 400 ppm, p value < 0.05.

Gentamicin (GN) administration induced a significant renal impairment. A notable increase (*p* < 0.05) in serum urea and creatinine levels. In parallel, it caused considerable oxidative stress in renal tissues, reflected by a significant (*p* < 0.05) increase in MDA and nitrite levels; a decrease in the activity of SOD compared with the control group, as shown in Figs. 7 and 8. The nephroprotective potential of MU-50 against gentamicin-induced toxicity can be explained by both direct and indirect antioxidant mechanisms, as illustrated in Fig. 8. Phenolic compounds not only scavenge reactive oxygen species but also upregulate significantly (*p* < 0.05) endogenous defense enzymes such as superoxide dismutase (SOD) and catalase (CAT), preserving renal tissue integrity. The observed decreases in serum urea, creatinine, and tissue MDA, together with restored SOD activity and improved histological appearance, support the extract’s ability to mitigate oxidative and inflammatory stress in renal tissues.

**Fig. 9:**
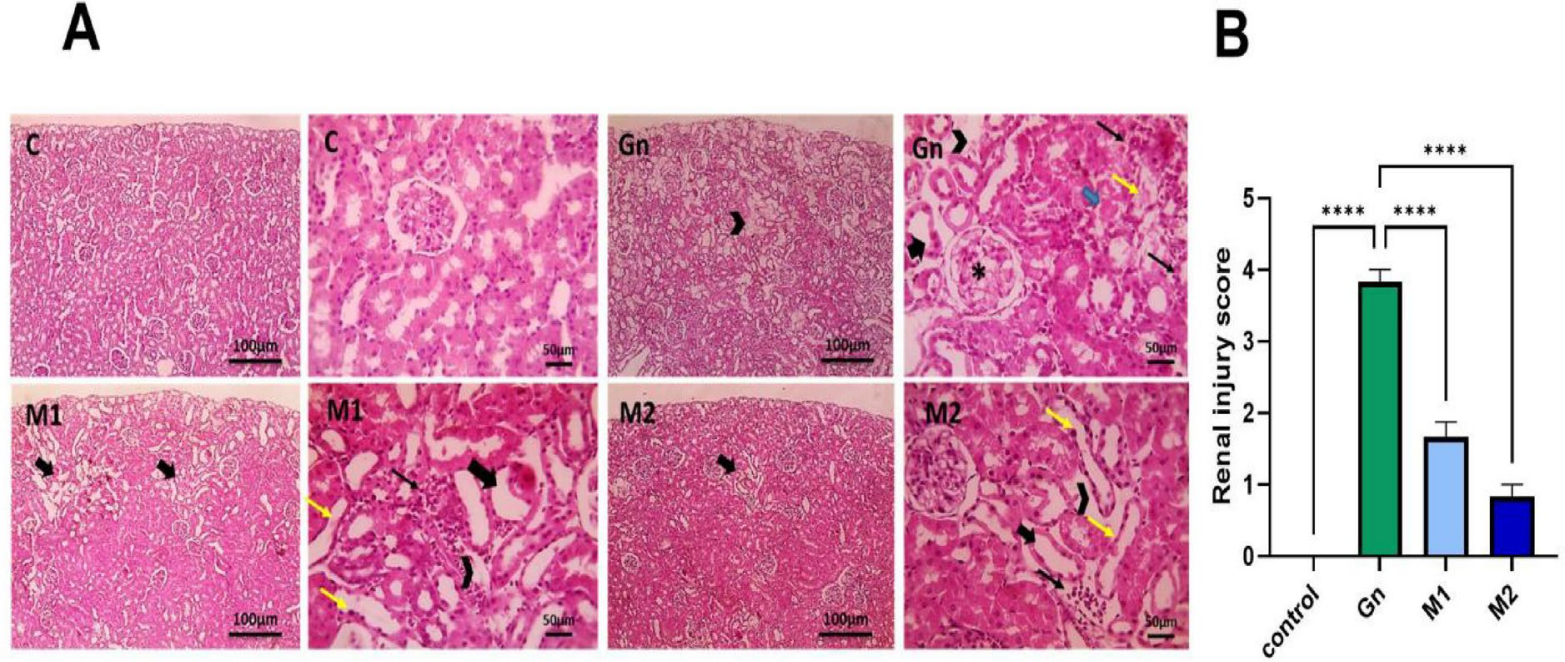
Photomicrographs of H & E-stained kidney sections from control, Gn, and different phenol extracts and histopathological scoring of renal injury. Thick black arrows: tubular dilation. Yellow arrows: atrophy. Thick blue marrows: necrosis. * Degenerated glomerular tuft. Arrowheads: interstitial edema. Low magnification X: 100 bar 100 and high magnification X: 400 bar 50. Renal injury scores are statistically analyzed by Kruskal-Walli’s test followed by Dunnk’s test to compare all means. Data are represented as mean ± SEM. Different alphabetical letters mean significant at *p>*0.05. C: Control, Gn: Gentamycin, M: Moringa, n=6/group.

Histopathological findings confirmed the biochemical results. Kidney sections from control animals displayed normal glomerular and tubular architecture (Fig. 9, C), while those from GN-treated rats exhibited extensive damage, including tubular dilation, necrosis, glomerular tuft degeneration, and interstitial edema with mononuclear cell infiltration (Fig. 9**,** GN). Rats treated with M_1_ showed multifocal tissue injury characterized by tubular atrophy, dilation, and mild interstitial inflammation (Fig. 9, M1). Conversely, M_2_-treated rats exhibited only focal lesions and preserved overall renal structure. It indicated a stronger protective nephrotoxicity effect^13,24^ (Fig. 9, M2). These histological findings were validated by morphometric analysis, which demonstrated a considerable decrease in kidney damage scores in both therapy groups, particularly in the M_2_ group, as seen in Fig. 9. Our in vivo results further extend previous work on *Moringa oleifera* extracts and nephroprotection^13,24^. It was demonstrated that this strategy resulted in more pronounced improvements in key biochemical markers and histopathological parameters.

### Physicochemical and phytochemical profile of MU-50 orange juice

In contrast to previous studies that primarily explored the use of *Moringa oleifera* extracts in meat products^46^ or evaluated their phytochemical properties in isolation^20,44^. Our work represents one of the first attempts to fortify orange juice with ultrasound-assisted *Moringa oleifera* leaf extract (MU-50) at 200 and 400 ppm as well as evaluate its physicochemical, microbiological, antioxidant, and sensory characteristics in parallel with in vivo nephroprotection (Table 5). Orange juice total soluble solids (TSS) remained stable. Titratable acidity was increased slightly, which may have contributed to a more pronounced tartness. Orange juice’s color revealed a visible shift towards a small, darker hue with a subtle greenish tint at the higher extract concentration. It is related to the moringa leaves’ chlorophyll content. Juice’s color changes were similar to those reported by Ahmed^77^, who noted a similar greenish tint in moringa fortified smoothies, but the intensity of the hue in the current study was less pronounced. This may be due to the use of the extract concentration as a powder.

**Table 5:** Effect of fortification on the physicochemical, color, and microbiological properties of orange juice

Orange Juice	Parameters	Control	200 ppm	400 ppm
Physicochemical and phytochemical Properties	TSS (°Brix)	9^a^	9^a^	9^a^
	Total Acidity (% citric acid)	1.31^a^	1.34^a^	1.36^a^
	pH	3.54^a^	3.52^a^	3.51^a^
	Vitamin C (µg / ml)	569.7 ± 0.01^c^	575.8 ± 0.05^b^	598.9 ± 0.03^a^
	β- carotene (µg / g)	2.74 ± 0.06^c^	3.42 ± 0.02	4.23 ± 0.02
	DPPH(%)	56.5 ± 0.2^c^	59.7 ± 0.04^b^	60.9 ± 0.12^a^
Color Attributes	L* (Lightness)	39.9 ± 0.14^a^	39.7 ±0.17^ab^	39.0 ± 0.04^b^
	a* (Red–Green)	-4.76 ± 0.66^c^	-3.75 ±0.18^b^	-3.54 ± 0.04^a^
	b* (Yellow–Blue)	27.5 ± 0.58^a^	26.9 ± 0.69^ab^	26.5 ± 0.1^b^
	h° (hue angle)	96.5 ± 0.47^a^	95.9 ± 0.7^ab^	95.5±0.5^b^
Microbiological Properties	Total Plate Count (log CFU/ml)	8.3*10^3^^a^	3.5*10^3^^b^	2.9*10^3^^c^
	Yeast & Mold (log CFU/ml)	5*10^a^	3*10^b^	1*10^c^
	Coliforms (MPN/ml)	nd*	nd*	nd*

*The data were obtained from three triplicate measurement (n = 3) and presented as the mean ± standard deviation (SD). The statistical significances are denoted as *p > 0.05*.; nd: not detected

Phytochemically, the inclusion of different levels of ultrasound-assisted moringa extract in orange juice showed significant variations (*p* < *0.05*) in free radical scavenging ability. Although polyphenols are heat-sensitive, the mild pasteurization applied in this study (90°C for 30 s) was unlikely to cause major degradation. Instead, short thermal exposure can facilitate the release of bound or glycosylated phenolics, resulting in more active aglycone forms. Additionally, the orange juice matrix, rich in sugars, organic acids, and ascorbic acid, may protect phenolic compounds through stabilizing interactions and antioxidant synergy. These factors collectively explain the preserved or enhanced antioxidant activity of the fortified juice after pasteurization. Vitamin C content was significantly higher in the fortified juices, with the 400-ppm concentration showing the most notable increase. This finding may outperform that of Richa^78^, who showed an increment in fruit beverages’ polyphenols supplemented with moringa leaves but did not achieve the same enhancement in vitamin C content. β-Carotene was increased in fortified juice about 1.5-fold more than control (Table 5). Conversely, some studies did not directly assess β-carotene content, although they focused on ultrasonic extraction at 40°C for 20 min to enhance total phenolic and flavonoid contents from *Moringa stenopetala*^45,79^. The tiny adjustment in hue angle from 96.5° to 95.5° does not imply a significant color change. The juice’s original orange look was expertly preserved by adding extracts. Furthermore, one cup of fortified orange juice met the Recommended Daily Intake (RDI) for vitamins C and β-carotene.

**Fig. 10:**
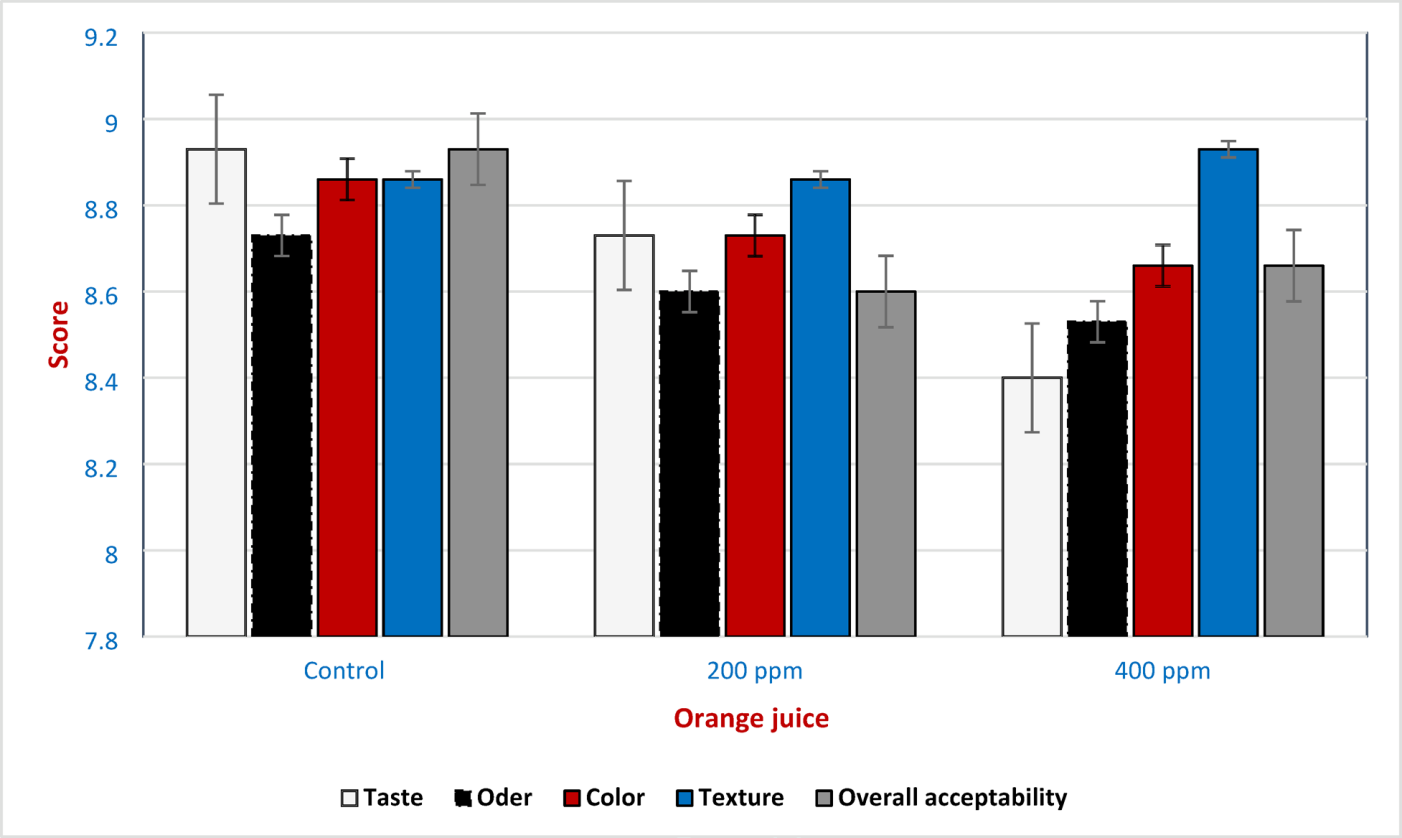
Organoleptic properties of fortified orange juices

Microbiologically, the inclusion of moringa extract at 400 ppm significantly reduced total plate count and yeast and mold populations. These effects may arise from phenolic–matrix interactions that protect active compounds from degradation and interfere with microbial enzymes or membrane permeability^80^. The extract’s stability under mild pasteurization suggests that its phenolics, particularly glycosylated derivatives, possess sufficient thermal resistance or are released in more active forms during short-time heating^81^. Sensory analysis showed that incorporating moringa extract at 200 ppm and 400 ppm concentrations had no negative impact on the juice’s overall acceptability. Panelists rated all samples as acceptable in terms of taste, aroma, appearance, and overall preference (Fig. 10). This result is contrary to studies by Trigo^82^, who reported that negative sensory effects were observed in moringa-fortified food matrices.

## Conclusion

This study has a dual evaluation integrating biological efficacy with a practical food matrix, which provides a translational dimension absent from earlier methods and highlights the potential of *Moringa oleifera* extracts as functional ingredients in commercial beverages and nutraceutical industries. The optimized 50% hydroethanolic extract (MU-50) exhibited superior antioxidant activity and provided significant protection against gentamicin-induced nephrotoxicity in a rat model. Comprehensive FTIR, GC–MS, and HPLC investigations revealed the existence of key phenolic components responsible for these effects. Fortified orange juice provides the Recommended Daily Intake (RDI) for vitamin C and β-carotene. Additionally, it demonstrated markedly increased antioxidant activity, reduced microbial load, and remained within consumer acceptable ranges. It is important to recognize that, this study has certain limitations, longer-term and dose–response studies are advised to confirm safety and efficacy. Future research should be also evaluating the scalability and storage durability of adding extracts to different food matrices. Addressing these characteristics would improve *Moringa oleifera’s* translational potential as a sustainable source of functional bioactive components.

## Data Availability

The datasets utilized and analyzed during this investigation are available upon reasonable request from the corresponding author**.**
