# Correction: Ultrasound assisted extraction enhances phytochemical profile and functional properties of moringa leaf extract with protection against gentamicin induced nephrotoxicity

**DOI:** 10.1038/s41598-026-54647-1

**Published:** 2026-06-04

**Authors:** Asmaa M. Shehata, Sanaa M. Abdel-Hameed, Aliaa F. Anter, Rokaia R. Abdelsalam

**Affiliations:** 1 Food Science Department, Faculty of Agriculture, Minia University, Minya, 61519 Egypt; 2 Pharmacology and Toxicology Department, Faculty of Pharmacy, Minia University, Minya, 61519 Egypt

Correction to: *Scientific Reports* 10.1038/s41598-025-27520, published online 26 November 2025

The original version of this Article contained an error in Fig. 6A, where an incorrect histopathological image panel was included during figure preparation. The incorrect Figure 6 along with its captions is provided below.

**Fig. 6:**
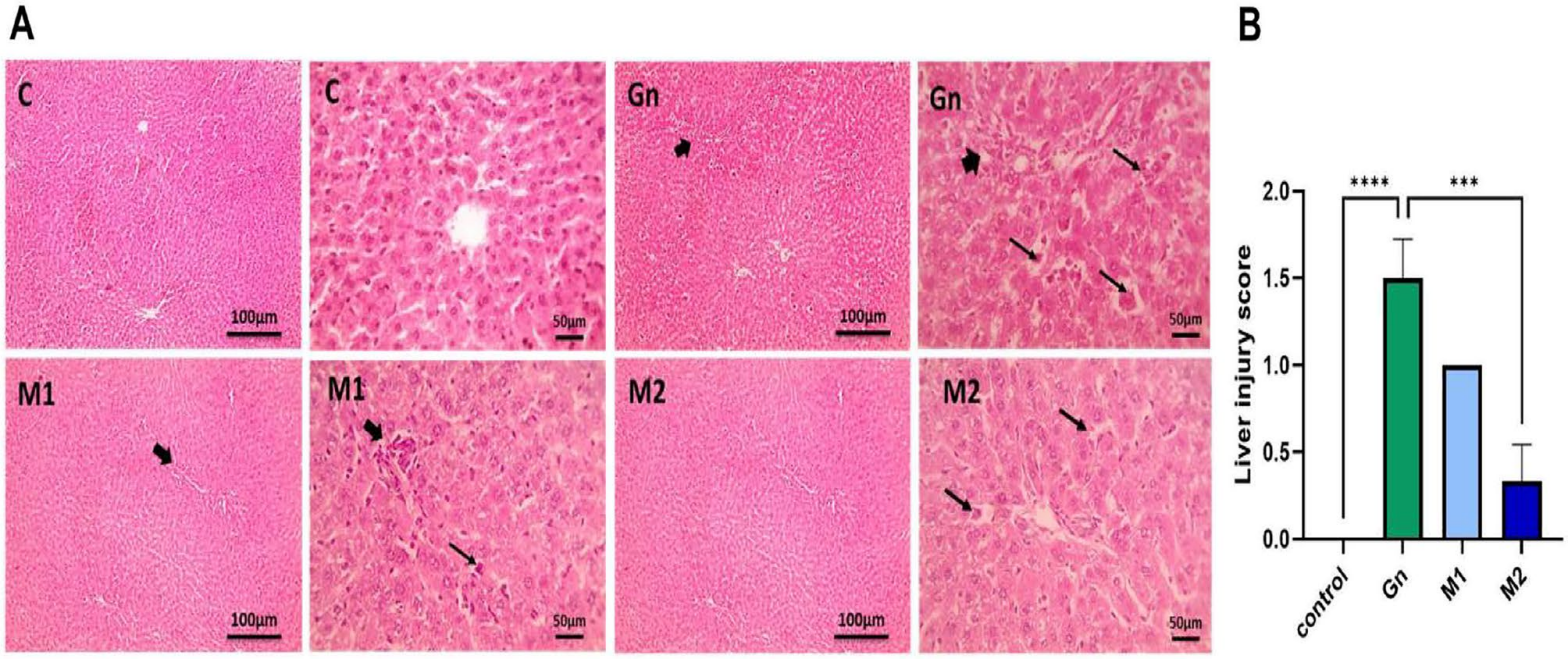
Photomicrographs of H & E-stained liver sections from control, GN, and different phenol extracts and histopathological scoring of hepatic injury. Thick black arrows: portal inflammation. Thin black arrows: extramedullary hematopoiesis in dilated sinusoids. Low magnification X: 100 bar 100 and high magnification X: 400 bar 50. Hepatic injury scores are statistically analyzed by Kruskal-Walli’s test followed by Dunn’s test to compare all means. Data are represented as mean ± SEM. Different alphabetical letters mean significant at *p* > 0.05. C: Control, Gn: Gentamycin*,* M: moringa*,* n = 6/group.

The original Article has been corrected.

